# Comparison of the Effectiveness of an Abbreviated Program versus a Standard Program in Mindfulness, Self-Compassion and Self-Perceived Empathy in Tutors and Resident Intern Specialists of Family and Community Medicine and Nursing in Spain

**DOI:** 10.3390/ijerph18084340

**Published:** 2021-04-20

**Authors:** Luis Ángel Pérula-de Torres, Juan Carlos Verdes-Montenegro-Atalaya, Elena Melús-Palazón, Leonor García-de Vinuesa, Francisco Javier Valverde, Luis Alberto Rodríguez, Norberto Lietor-Villajos, Cruz Bartolomé-Moreno, Herminia Moreno-Martos, Javier García-Campayo, Josefa González-Santos, Paula Rodríguez-Fernández, Benito León-del-Barco, Raúl Soto-Cámara, Jerónimo J. González-Bernal, The MINDUUDD Collaborative Study Group

**Affiliations:** 1 Multi-Professional Teaching Unit for Family and Community Care of Córdoba, Healthcare District of Córdoba and Guadalquivir, Institute Maimónides of Research Córdoba (Imibic), Reina Sofía University Hospital, University of Córdoba, 14001 Cordoba, Spain; langel.perula.sspa@juntadeandalucia.es; 2 Family and Community Medicine Teaching Department of Burgos, 09006 Burgos, Spain; 3 Family and Community Medicine Teaching Department of Zaragoza Sector 1, 5018 Zaragoza, Spain; elenamelusp@gmail.com (E.M.-P.); cbartolomem@hotmail.com (C.B.-M.); 4 Multi-Professional Teaching Unit for Family and Community Care of Córdoba, Healthcare District of Córdoba and Guadalquivir, 14001 Córdoba, Spain; leonorgarciadevinuesa@gmail.com; 5 Family and Community Medicine Teaching Department of Jaen, 23007 Jaen, Spain; franciscoj.valverde.sspa@juntadeandalucia.es (F.J.V.); norberto.lietor.sspa@juntadeandalucia.es (N.L.-V.); 6 Family and Community Medicine Teaching Department of Ponferrada, Ponferrada, 24400 León, Spain; lalberto.rodriguez.sspa@juntadeandalucia.es; 7 Multi-Professional Teaching Unit for Family and Community Care of Almería, 04009 Almería, Spain; partaloa@gmail.com; 8 Psychiatry Department, Miguel Servet University Hospital, 50009 Zaragoza, Spain; jgarcamp@gmail.com; 9 Department of Health Sciences, University of Burgos, 09001 Burgos, Spain; prf0011@alu.ubu.es (P.R.-F.); jejavier@ubu.es (J.J.G.-B.); 10 Department of Psychology, Faculty of Teacher Training College, University of Extremadura, 10071 Caceres, Spain; bleon@unex.es

**Keywords:** mindfulness, self-compassion, empathy, tutors, resident intern specialists, MBSR

## Abstract

Health professionals are among the most vulnerable to work stress and emotional exhaustion problems. These health professionals include tutors and resident intern specialists, due to the growing demand for the former and the high work overload of the latter. Mindfulness training programs can support these professionals during times of crisis, such as the current global pandemic caused by the coronavirus-19 disease. The objective of this study was to compare the effectiveness of an abbreviated Mindfulness-Based Stress Reduction (MBSR) and Mindful Self-Compassion (MSC) training program in relation to a standard training program on the levels of mindfulness, self-compassion, and self-perceived empathy in tutors and resident intern specialists of Family and Community Medicine and Nursing. A total of 112 professionals attached to six Spanish National Health System teaching units (TUs) participated in this randomized and controlled clinical trial. Experimental Group (GE) participants were included in the standard or abbreviated MBSR programs. The Five Facet Mindfulness Questionnaire (FFMQ), the Self-Compassion Scale short form (SCS-SF), and the Jefferson Scale of Physician Empathy (JSPE) were administered three times during the study: before, immediately after, and 3 months after the intervention. Adjusted covariance analysis (ANCOVA), using pretest scores as the covariate, showed a significant increase in mindfulness (F_(2,91)_ = 3.271; *p* = 0.042; η^2^ = 0.067) and self-compassion (F_(2,91)_ = 6.046; *p* = 0.003; η^2^ = 0.117) in the post-test visit, and in self-compassion (F_(2,79)_ = 3.880; *p* = 0.025; η^2^ = 0.089) in the follow-up visit, attributable to the implementation of the standard training program. The standard MBSR and MSC training program improves levels of mindfulness and self-compassion, and promotes long-lasting effects in tutors and resident intern specialists. New studies are needed to demonstrate the effectiveness of abbreviated training programs.

## 1. Introduction

Health professionals are exposed to stressful and emotionally intense situations in the workplace, which makes them more vulnerable to problems of work stress and emotional exhaustion [1,2]. This is particularly the case for the group comprising tutors and resident specialists in internships [3].

Consideration of the correct functioning and effective management of workers and organizational groups is essential to promote good work performance, in addition to improving psychosocial well-being and increasing the quality of work life of employees [4,5,6,7,8]. A wide variety of studies have investigated the effect of different instruments and techniques on the psychological functioning of healthcare professionals, showing positive results in attention, self-compassion, anxiety, emotional exhaustion, stress, and rumination [9,10,11,12,13], but there is no specific action protocol to prevent and treat psychological and emotional symptoms originating in the workplace [10]. In Spain, the implementation of different strategies to deal with this problem has been uneven in recent years [14], focusing mainly on interventions to improve or support individual coping skills [15,16]. In addition, several studies have reported the need to provide interventions to support the mental health of these professionals during the early stages of the global pandemic caused by COVID-19 [17,18]. 

The regular practice of mindfulness or meditation interventions has been suggested by various authors for this purpose [19,20], and these practices have achieved positive results in coping with stress, burnout, empathy, and satisfaction levels of health professionals [15,16,21,22], in addition to patient outcomes [23]. Although various definitions of mindfulness exist, it can be defined as the quality of awareness that is produced by intentionally focusing on present moment experiences in an accepting and non-judgmental way [24]. Mindfulness is one of the most widely used meditation techniques today, with a high reputation in Western countries [25]. This is a third generation therapy that involves self-regulation of attention to the experience of the present moment [26]. Jon Kabat-Zinn developed the best-known mindfulness program to date at the University of Massachusetts, i.e., the Mindfulness-Based Stress Reduction (MBSR) program [27]. It consists of eight weekly group sessions, lasting 120–150 min, and 45 min of daily practice at home [28,29,30]. Due to the high degree of commitment and adherence required, the possibility of reducing its duration is being studied to guarantee accessibility and achieve greater viability, while maintaining its effectiveness at all times [31,32,33,34]. 

Although the effectiveness of various mindfulness programs on the stress and burnout levels of healthcare workers has been demonstrated [15,16,19,20,21,22], this technique involves being in contact with one’s own painful thoughts and feelings by observing and accepting them as they are [25]; therefore, aspects such as empathy, self-awareness and self-compassion should also be considered when studying the effectiveness of these programs [15]. 

The majority of previous studies do not consider mindfulness to be a primary outcome, making it difficult to determine whether treatment results were caused by increased mindfulness skills or other factors, such as social support or group cohesion [35,36]. Mindfulness is a process that requires observing, describing, and acting conscientiously, without judging [37]. Healthcare professionals with developed self-awareness or full attention have a higher level of self-care and greater ability to engage with patients, without experiencing additional stress [38]. 

Empathy is the general ability of a person to resonate with the emotional states of others, and can lead to compassion or empathetic distress depending on how subjects respond to the suffering of others [39]. Compassionate responses are positive feelings towards the other and involves prosocial behavior, whereas empathetic distress refers to negative feelings associated with avoidance or flight [40,41]. Self-compassion also involves assertively managing one’s emotions [41], and makes people adopt a growth mindset and set goals related to learning and personal growth. Training programs exist to help achieve this feeling, such as Mindful Self-Compassion (MSC) [42], which has been shown to reduce the levels of work stress of healthcare professionals, and to improve professional–patient communication, and clinical and psychological parameters of the patient [41]. 

The effects of mindfulness and self-compassion practice have been widely studied in relation to burnout, but new studies are needed to consider other relevant psychological variables in healthcare workers and to analyze the maintenance of its effects in the medium- to long-term [31,32,38]. Furthermore, despite the existence of scientific evidence supporting the effectiveness of abbreviated MBSR training programs [43], their effectiveness compared to standard training programs among healthcare professionals should be further investigated. In this way, these practices could be included in continuing training curriculum programs. Therefore, the objective of this study was to compare the effectiveness of an abbreviated MBSR and MSC training program in relation to a standard training program on the levels of mindfulness, self-compassion, and self-perceived empathy in tutors and resident intern specialists in Family and Community Medicine and Nursing, attached to six teaching units (TUs) of the Spanish National Health System.

## 2. Materials and Methods

### 2.1. Design and Setting

The design of this study corresponds to an open-label, pragmatic, non-inferiority, multicenter, cluster, controlled, and randomized clinical trial with three parallel arms: control group (CG), intervention group 1 (EG1), and intervention group 2 (EG2). 

The protocol of this clinical trial was previously published [44] and registered in ClinicalTrials.gov website, dependent on the U.S. National Library of Medicine, with reference number NCT03629457. 

The results reported in this manuscript refer to the levels of mindfulness, self-compassion, and self-perceived empathy of tutors and resident intern specialist, as the main variables analyzed in this study.

### 2.2. Study Participants and Recruitment

The study population consisted of all tutors (*n =* 297) or resident intern specialists in Family and Community Medicine or Nursing (*n =* 595), who were working in one of the Health Centers attached to the following 6 TUs of the Spanish National Health System: Cordoba (*n =* 256), Almeria (*n =* 147), Jaen (*n* = 185), Burgos (*n =* 64), Ponferrada (*n* = 63), and Zaragoza Sector I (*n =* 87). Exclusion criteria considered were having previously attended to a mindfulness training course or workshop with a minimum duration of 4 weeks, practicing this technique regularly and actively, being in prolonged sick leave during fieldwork, or suffering from any mental disorder that hindered the understanding and subsequent development of interventions.

Through the existing communication channels in each of the 6 TUs, all possible participants in the study were contacted. A first face-to-face meeting was held in which the objective and methodology of the research, and its voluntary nature, were explained to them. In addition, they were invited to participate in the study, and required to sign a commitment form and give their written informed consent in case of acceptance.

### 2.3. Sample Size

The sample size was estimated based on the potential modification of the main variable, i.e., the score of the Five Facet Mindfulness Questionnaire (FFMQ). To detect a minimum difference of ≥15 points in the FFMQ between CG and EGs, 114 participants (38 per group) was required, assuming an alpha risk of 0.05, a beta risk of 0.20, in two-side contrast, and a standard deviation (SD) of ±20 points. A predicted drop-out rate of 25% during follow-up phase was assumed [45]. This estimate took into account the results obtained in a similar previous study [20]. In addition, factors such as the type of study or its design were also taken into account when the sample size was calculated. In this calculation, a multiplying factor was applied that allowed the same power to be achieved between the intergroup and intragroup variance [46]. Based on this, and taking into account an intragroup correlation coefficient <0.05, which is the most common coefficient value in clinical trials developed in Primary Care [47], and an effect of the design type of 1.7, it was concluded that the sample should be made up of 132 professionals, 44 in each comparison group and 22 for each TU. 

### 2.4. Procedure and Randomisation

One week before the start of the sessions in the EGs, all participants attended a first initial or baseline evaluation visit (pre-test), in which the study variables were measured. Subsequently, 4 weeks after the initial evaluation visit for EG1 participants and 8 weeks for those of EG2 and CG, the same variables were assessed again at the final evaluation visit (post-test). Furthermore, EG1 and EG2 participants had to attend to a third evaluation visit (follow-up), 3 months after the end of the intervention program, to assess the maintenance of its effect in the medium-long term. 

**Figure 1:**
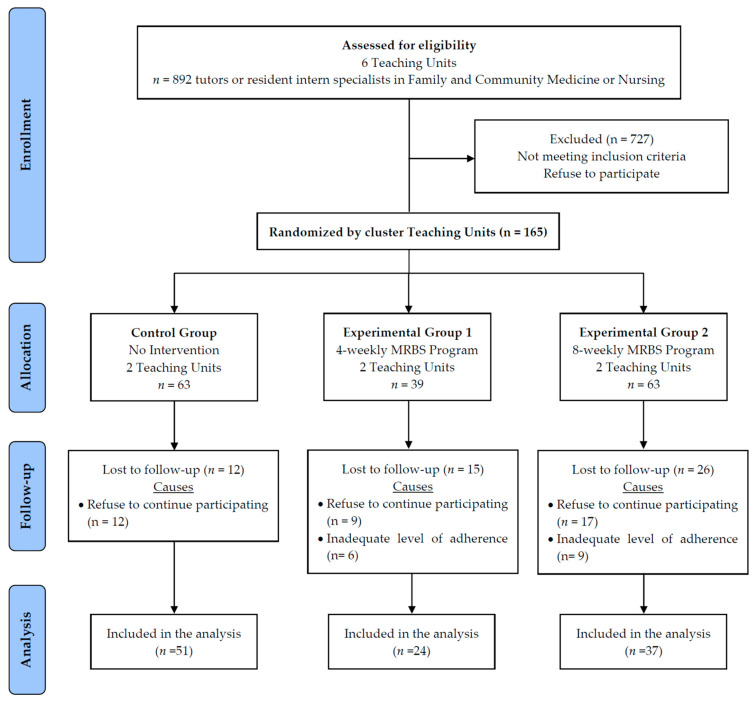
Flow chart of participants through the study.

The process of randomization of healthcare professionals in the different study groups was carried out by clusters. Each of the 6 TUs analyzed was considered as a different and independent cluster, so that its participants were assigned to CG (2 TU), EG1 (2 TU), or EG2 (2 TU). In addition, the participants from each TU were also stratified according to the type of professional (66 tutors versus 66 resident intern specialists) (Figure 1).

During the development of the study, it was not possible to blind all participants due to the characteristics of the interventions. However, in order to minimize cross-contamination between groups, both the researcher who conducted the evaluation visits and the one who carried out the statistical analysis of the data remained blinded to the group to which the participants belonged. In turn, all participants received clear instructions to not reveal the group to which their TUs had been randomly assigned to blinded researchers during evaluation visits.

### 2.5. Intervention

All EG1 and EG2 participants were included in an MBSR training program [31,32], complemented by Mindful Self Compassion [48,49,50] practices. The sessions were adapted to the characteristics of each group, differing only in the duration and time dedicated to the different tasks by the participants [31,32]. In EG1, participants received 4 sessions per week, 2.5 h long, which were complemented by the daily practice of 15 min at home (abbreviated program); while at EG2, participants received 8 sessions per week, lasting 2.5 h, along with the practice of 30 min a day at home (standard program). At all times, the practical application of group sessions in the personal and/or professional fields of the participants was looked for. To this end, moments of silence were alternated with other moments of collective exploration on the best strategies to address complex and difficult situations of their development. Some of the aspects covered were the knowledge of mindfulness, the perception of reality, stress and emotional management, the use of conscious communication, resilience, self-care or time management, and the integration of mindfulness into daily life. The activities developed in each of the sessions were detailed previously in the study protocol [44]. To avoid any variability associated with the therapist, all sessions were taught by the same accredited instructors, following standardized and uniform methodological criteria.

The participants included in the CG did not receive any type of intervention. In the initial evaluation visit, they had to pledge not to participate in any theoretical–practical session of mindfulness or meditation during the period of development of the study. Once the fieldwork was completed, they were offered the opportunity to participate in an abbreviated training program.

### 2.6. Main Outcomes

Mindfulness, self-compassion, and self-perceived empathy were the main outcomes of the study, which were assessed in the different evaluation visits (initial, final, follow-up).

Mindfulness was measured using the FFMQ, validated in the Spanish population by Cebolla et al. [51]. This self-report consists of 39 items, distributed in 5 subscales: observing, describing, acting with awareness, non-judging of inner experiences, and non-reactivity to inner experience. For example, one of the items to assess is: “When I´m walking, I deliberately notice the sensations of my body moving”. Each participant must indicate their agree or disagreement with the content of the statement using a five-point Likert scale, where 1 corresponds to “never or very rarely true”, and 5 to “very often or always true”. The total score ranges from 39 to 195 points, with higher scores indicating more mindfulness [52,53,54]. This scale has an adequate level of internal consistency, with a Cronbach alpha ranging from 0.75 to 0.92 ([51]; a score of 0.84 was obtained in this study).

The Self-Compassion Scale short form (SCS-SF) was used to assess how the subject usually acts towards themselves in difficult times [55]. This questionnaire, validated by García-Campayo et al. [56] in the Spanish population, consists of 12 items, distributed in 6 subscales: self-friendship, common humanity, mindfulness, self-judgment, isolation and over identification. For example, one of the items to assess is: “When I fail at something important to me I become consumed by feelings of inadequacy”. Each item is valued using a six-point Likert scale, where 0 corresponds to “almost never” and 5 to “almost always”. The total score ranges from 0 to 60 points, with higher scores indicating more self-compassion [55,56]. This scale has an adequate level of internal consistency, with a Cronbach alpha ranging from 0.71 to 0.77 [56]; a score of 0.88 was obtained in this study.

Self-perceived empathy was assessed by the Jefferson Scale of Physician Empathy (JSPE), which was translated, adapted, and validated in the Spanish population by Blanco et al. [57,58]. Through its 20 items and 7 possible responses, this scale analyzes three dimensions of empathy: taking perspective or cognitive empathy, compassionate attention or emotional empathy, and “ability to put oneself in the patient’s shoes”. One example of the items assessed in this scale is: “My understanding of how my patients and their families feel is not a relevant factor for medical treatment”. The total score ranges from 20 to 140 points, with higher values indicating more empathy. This scale has an adequate level of internal consistency, with a Cronbach alpha of 0.89 [57,58]; a score of 0.70 was obtained in this study.

Participants’ adherence to the training programs was monitored by checking attendance at the different group sessions and by the personal self-registration of the practices carried out at home. An adequate level of adherence was considered when the participant had completed at least 3 of the 4 face-to-face sessions in EG1, or 6 of the 8 in EG2. Only data from participants with an adequate level of adherence were included in the subsequent statistical analysis.

To control the predictive or confusion effect, the following socio-demographic information was collected from the participants in the initial evaluation visit: age, sex (male or female), professional category (physician or nurse), type of professional (tutor or resident intern specialist), work center (hospital or health center), time working in the Spanish National Health System or TU.

### 2.7. Data Collection Procedure, Data Management and Monitoring

In each evaluation and follow-up visit, the data was collected by a researcher, who had been previously trained for this task. This person did not participate in the randomization process or in the subsequent statistical analysis of the data. Each participant was identified through a unique alphanumeric code to ensure the data referred to the same person in the different evaluation visits. A database was created for this purpose, which could only be accessed by the researchers responsible for statistical analysis. To minimize the rate of data errors in the recording, a double entry procedure was used in each of the questionnaires. The principal investigator of the study was responsible for deleting the data once the study was completed.

### 2.8. Ethical Considerations

The study was approved by the Ethics and Clinical Research Committee of the Reina Sofia University Hospital in Córdoba (Spain), with reference number 3845. According to the Helsinki Declaration, all participants were informed of the objectives of the project, and its potential risks and benefits of the evaluations to be conducted. Each subject was required to provide the signed informed consent for his/her inclusion in the study. The data obtained were not used for purposes other than those expressed in the informed consent. The confidentiality of the participants’ data was guaranteed at all times in accordance with the provisions of the Organic Law 3/2018, of 5 December, on Personal Data Protection and Guarantee of Digital Rights, the Law 14/2007, of 3 July, on Biomedical Research, and the EU Regulation 2016/679 of the European Parliament and of the Council of 27 April 2016 on the General Data Protection of Natural Persons with regard to the Processing of Personal Data and on the Free Movement of such Data.

### 2.9. Statistical Analyses

To minimize and control the effects of non-random drop-outs and losses, an intent-to-treat analysis was performed. In the descriptive analysis of the sample, mean and standard deviation (SD) were used in the case of quantitative variables, or absolute frequencies and percentages for categorical variables. Compliance with the normality criteria in quantitative variables was verified using the Kolmogorov–Smirnov test. In those cases in which these criteria were not respected, median and interquartile range (IQR) were calculated. To assess the comparability of the groups in the initial evaluation visit in terms of age, gender, professional category, type of professional, work center, or time working in the Spanish National Health System, the chi-square test and the Student´s t-test for independent samples were used, or their corresponding non-parametric tests. A one-way variance analysis (ANOVA) was used to analyze the effect of the intervention on mindfulness, self-compassion, and self-perceived empathy in CG, EG1 and EG2 participants. A repeated measures ANOVA was used to compare differences in outcomes in mindfulness, self-compassion, and self-perceived empathy between groups before and after the interventions. The Bonferroni post hoc test was used to determine between which groups there were differences. The effect size of the interventions was estimated using the squared eta coefficient (η^2^), interpreted according to the following criteria: if 0 ≤ η^2^ < 0.05, no effect; if 0.05 ≤ η^2^ < 0.26, the effect was minimal; if 0.26 ≤ η^2^ < 0.64, the effect was moderate; and if η^2^ ≥ 0.64, the effect was strong [59]. Finally, to eliminate from the dependent variables (post-test and follow-up scores) the effect attributable to variables not included in the design and, therefore, not subjected to experimental control, a covariance analysis (ANCOVA) was performed, using the pre-test scores of the dependent variables as the covariate and the intervention groups as a fixed factor. Statistical analyses were performed with SPSS version 25.0 (IBM SPSS Inc., Chicago, IL, USA) and MLwiN version 3.00 software (Centre for Multilevel Modelling, University of Bristol: Bristol, UK, 2019). Statistical significance was considered if *p* < 0.05. 

## 3. Results

### 3.1. Baseline Characterists of the Study Participants

The study sample consisted of 165 subjects, of which 63 were assigned to CG, 39 to EG1, and 63 to EG2. There were 53 losses in the follow-up phase, 38 because the subject refused to continue participating in the study and 15 due to an inadequate level of adherence to the training program. Therefore, 112 participants completed the study and were included in the analysis, with 51 in the CG, 24 in the EG1, and 37 in the EG2. (Figure 1).

Table 1 summarizes the baseline socio-demographic characteristics of participants according to the study group. Women represented 76.79% of the participants (*n* = 86), with a mean age of 40.61 years (SD ± 12.61). Most of the participants worked in Primary Care (*n* = 95; 84.82%), with the physician being the most represented professional category (*n* = 95; 84.82%). The mean work experience was 12.88 years (SD ± 13.15). The sample was distributed equally to tutors and resident intern specialist (50 versus 62). Statistically significant differences were observed between the three groups in age, professional type, and work experience.

### 3.2. Mindfulness, Self-Compassion and Self-Perceived Empathy

Table 2 summarizes the differences between CG, EG1, and EG2 in levels of mindfulness, self-compassion, and self-perceived empathy. No statistically significant differences were observed between the groups, so all participating TUs were equivalent and started from the same situation. In the post-test and follow-up visits, statistically significant differences were observed in the mindfulness and self-compassion scores of CG participants with respect to those of EG2, with this latter group showing higher levels. In all cases, the effect size was significant and weak (η^2^ ≤ 0.076). No statistically significant differences were obtained in levels of self-perceived empathy between the study groups in any of the evaluations. 

When comparing the FFMQ, SCS, and JSPE scores obtained by each of the groups analyzed at the three time points studied, no statistically significant differences were found between CG participants. Subjects who participated in the abbreviated training program demonstrated a significant increase in the JSPE score in the follow-up visit compared to the pre-test visit, with a weak effect size (η^2^ = 0.223). Those participants who were part of the standard training program obtained higher levels of mindfulness and self-compassion in the post-test and follow-up visits, with respect to the pre-test visit; however, unlike in the abbreviated training program group, no statistically significant differences in self-perceived empathy were observed. (Table 3).

**Figure 2:**
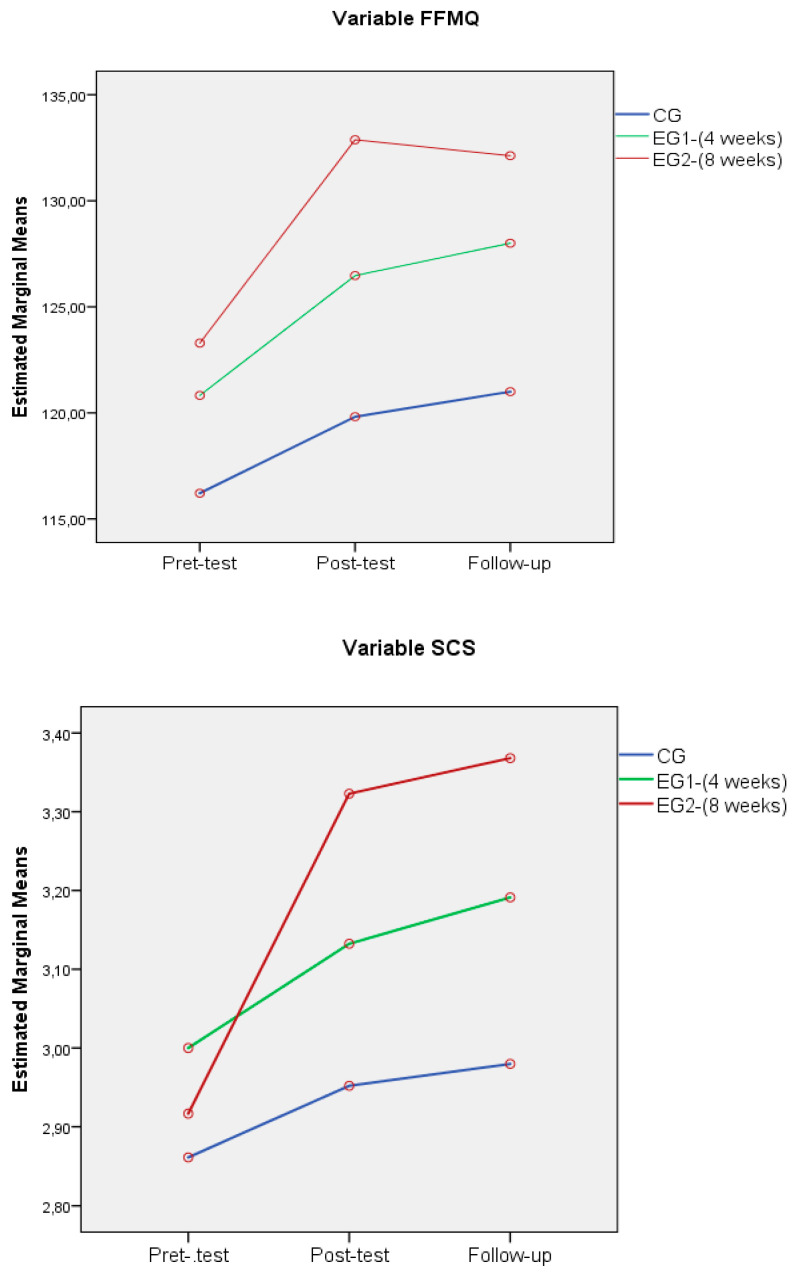
Line graphs of the FFQM and SCS scores of the different groups in the three temporal points.

In the following line graphs, variables with statistically significant differences between the scores of the different groups in the three temporal points are represented (Figure 2).

ANCOVA showed statistically significant differences between CG and EGs in the mindfulness and self-compassion in the post-test visit, confirming the results observed in the previous intergroup comparisons (Table 4). Therefore, these differences, mainly in EG2, could be attributed to the performed intervention.

In the same way, the significant differences found in the self-compassion between the CG and the EGs in the follow-up visit could be attributed to the intervention carried out, based on the results obtained from ANCOVA (Table 5).

## 4. Discussion

The main findings of this study showed that there was no significant improvement in mindfulness, self-compassion, and self-perceived empathy in the tutors and resident intern specialists who received the abbreviated mindfulness training program. The participants of the standard training program improved their levels of mindfulness and self-compassion, effects that were maintained over time; however, no significant impacts were observed on levels of self-perceived empathy.

Structured and regular mindfulness or meditation trainings have been show to improve emotional regulation, and reduce anxiety, depression, and post-traumatic stress disorder [60,61,62], which are symptoms associated with COVID-19, especially in health professionals [63]. In times of crisis such as the current global pandemic, these techniques have been adapted using different apps and online e-Health and Telehealth, although new studies are needed to demonstrate their effectiveness [64,65]. 

Previous research has shown that mindfulness training programs such as MBSR significantly increase self-compassion, which is essential to reduce the stress levels experienced by healthcare professionals [43,66,67,68]. The importance of implementing training programs that address these two aspects is increasingly evident; however, most published studies focus on mindfulness or self-compassion independently [69,70]. 

The combination of mindfulness programs and empirical support training to cultivate self-care skills improves participants’ self-compassion, full attention, and interpersonal conflict [71,72,73]. A study by Keng et al., whose objective was to examine the independent role of mindfulness and self-compassion as potential mediators of the effects of an MBRS program on various processes and behaviors related to the regulation of emotions, showed that self-compassion training reduced cognitive trends of mis-adaptive coping and increased willingness to accept and experience new emotions [67].

In line with the above, Shapiro et al. designed an intervention based on the conventional MBSR training program combined with guided compassion meditation, and observed a significant increase in self-compassion in EG compared to CG (22.0% versus 3.0%). These results are consistent with those obtained in the present study, in which participants who received the standard MBSR and MSC training program reported significantly higher levels of self-compassion than CG professionals [66].

Krasner et al. assessed the effect of a mindfulness and self-compassion training program on the exhaustion, empathy, and mood of Primary Care physicians. The results of this study demonstrated a significant increase in mindfulness skills and orientation, which was correlated with lasting improvements in exhaustion, mood disorders, and empathy [68]. In contrast to these findings, but in line with those obtained by Amutio et al. [74] and Galantino et al. [75], none of the training programs carried out in this study significantly increased the levels of self-perceived empathy of tutors and resident intern specialists. Empathetic ability enables a person to grasp and understand the feelings of others, although mismanagement can lead to compassion or empathetic distress [76]. The levels of self-perceived empathy have not increased in this study, which may be due to the fact that mindfulness training programs promote balanced levels of this aspect, counteracting their over-identification and reducing excessive fixation of negative thoughts [39,40,41]. 

This study provides information about the benefits of the standard MBSR and MSC training program in mindfulness, self-compassion, and self-perceived empathy in a group of the tutors and resident intern specialists in Spain. However, these findings should be considered within the context of their limitations. Although TUs were randomly assigned, with the aim of minimizing the risk of contamination, statistically significant differences were observed between the three groups in age, type of professional, and time working in the Spanish National Health System. Due to the epidemiological situation derived from the global pandemic caused by COVID-19, the final number of participants was lower than initially calculated, which may have influenced the results obtained. A selection bias may have occurred in this study because the characteristics of the non-responders might differ from those of the responders. To minimize and control this effect, an intention-to-treat analysis was performed. Although a representative sample of Spanish tutors and resident intern specialists in Family and Community Medicine or Nursing was available, the predominance of women, Primary Care workers, and physicians was able to condition the results obtained. In addition, although participants included in the CG were required to not participate in any theoretical–practical session of mindfulness or meditation, it was not possible to guarantee their inactivity during the fieldwork period, which could minimize the differences in the expected results when comparing this group with EGs. To verify that the effect of the interventions lasted over time, a follow-up assessment was made of the EG participants 3 months after their application. Ideally, this visit would have been delayed in time, which was not possible because some resident intern specialists completed their training period shortly after completing the study. These limitations must be taken into account because they may have influenced the results obtained in the study and reduced its representativeness.

Despite these limitations, this study is pioneering in comparing the effectiveness of a standard MBSR and MSC training program with an abbreviated program. Its main strengths are its longitudinal methodology, which allows determination of causal relationships between the study variables, the use of validated questionnaires for the Spanish population, which guarantees their validity and reduces the probability of information biases, in addition to the evaluation of effects over time.

## 5. Conclusions

The implementation of abbreviated mindfulness and self-compassion training programs for tutors and resident intern specialists in Family and Community Medicine or Nursing may be encouraging, due to their greater viability and accessibility. Nonetheless, more exhaustive and representative studies are required to support the effectiveness of these programs compared to the standard MBSR and MSC training programs, which have been shown to improve levels of mindfulness and self-compassion, and promote long-lasting effects in healthcare professionals.

## Figures and Tables

**Table 1:** Baseline characteristics of participants.

Variables	Total (*n* = 112)	CG (*n* = 51)	EG1 (*n* = 24)	EG2 (*n* = 37)	*p-*Value
**Age (years)**	41.61 ± 12.61	40.34 ± 13.22	47.66 ± 13.67	35.73 ± 12.04	<0.001
**Sex**					
Male	26 (23.21)	11 (21.57)	6 (25.00)	9 (24.32)	0.978
Female	86 (76.79)	40 (78.43)	18 (75.00)	28 (75.68)	
**Occupation**					
Physician	95 (84.82)	41 (80.39)	20 (83.33)	34 (91.89)	0.165
Nurse	17 (15.18)	10 (19.61)	4 (16.67)	3 (8.11)	
**Professional type**					
Tutor	50 (44.64)	24 (47.06)	15 (62.50)	11 (29.73)	<0.001
Resident	62 (55.36)	27 (52.94)	9 (37.50)	26 (70.27)	
**Work-place**					
Health Center	95 (84.82)	40 (78.43)	22 (91.67)	33 (89.19)	0.217
Hospital	17 (15.18)	11 (21.57)	2 (8.33)	4 (10.81)	
**Work experiences (years)**	12.88 ± 13.15	13.13 ± 12.95	19.49 ± 13.91	8.91 ± 11.06	<0.001

Values are expressed as mean ± standard deviation, median-interquartile range or frequencies (percentages). Abbreviations: CG: Control Group; EG1: Experimental Group, 4 weeks; EG2; Experimental Group, 8 weeks; SNS: National Health System.

**Table 2:** Comparison of FFQM, SCS, and JSPE according to the type of group using one-way ANOVA.

Evaluation	Variable	CG		EG1		EG2		F	*p*-Value	η^2^
		M	SD	M	SD	M	SD			
**Pre-test**	**FFMQ**	118.17	13.32	117.71	16.76	119.26	15.11	0.153	0.858	0.002
	**SCS**	2.88	0.73	2.97	0.88	2.82	0.93	0.381	0.683	0.005
	**JSPE**	124.14	8.53	120.12	15.98	122.44	12.72	1.281	0.281	0.016
**Post-test**	**FFMQ**	119.28 *	17.79	124.07	22.60	131.65 *	18.03	5.004	0.008	0.076
	**SCS**	2.98 *	0.89	3.15	0.98	3.47 *	0.74	3.789	0.025	0.058
	**JSPE**	124.35	8.40	120.85	15.74	126.85	9.06	2.634	0.076	0.041
**Follow-up**	**FFMQ**	121.03 *	18.29	125.04	22.62	131.97 *	18.22	3.461	0.035	0.060
	**SCS**	2.96 *	0.90	3.21	1.04	3.46 *	0.78	3.289	0.041	0.057
	**JSPE**	123.96	8.06	125.50	12.39	124.35	18.23	0.113	0.893	0.002

* *p*-value < 0.05 in post hoc analysis (Bonferroni), between CG and EG2. Abbreviations: CG: Control Group; EG1: Experimental Group, 4 weeks; EG2: Experimental Group, 8 weeks; M: mean; SD: standard deviation; FFMQ: Five Facet Mindfulness Questionnaire; SCS: Self-Compassion Scale; JSPE: Scale of Physician Empathy.

**Table 3:** Intra-group comparisons of FFQM, SCS and JSPE, using ANOVA for repeated measures.

Group	Variable	Pre-Test		Post-Test		Follow-Up		MS	F	*p*-Value	η^2^
		M	SD	M	SD	M	SD				
**CG**	**FFMQ**	118.17	13.32	119.28	17.79	121.03	18.29	205.283	2.009	0.143	0.059
	**SCS**	2.88	0.73	2.98	0.89	2.96	0.90	0.127	0.971	0.384	0.029
	**JSPE**	124.14	8.53	124.35	8.40	123.96	8.06	23.303	0.578	0.564	0.018
**EG1**	**FFMQ**	117.71	16.76	124.07	22.60	125.04	22.62	242.902	2.736	0.080	0.146
	**SCS**	2.97	0.88	3.15	0.98	3.21	1.04	0.163	1.160	0.326	0.068
	**JSPE**	120.12 *	15.98	120.85	15.74	125.50 *	12.39	240.137	4.584	0.018	0.223
**EG2**	**FFMQ**	119.26 *^,$^	15.11	131.65 ^$^	18.03	131.97 *	18.22	681.722	8.473	0.001	0.269
	**SCS**	2.82 *^,$^	0.93	3.47 ^$^	0.74	3.46 *	0.78	1.483	9.356	<0.001	0.289
	**JSPE**	122.44	12.72	126.85	9.06	124.35	18.23	245.847	1.596	0.214	0.065

^$^*p*-value < 0.05 in post hoc analysis (Bonferroni), between pre-test and post-test. * *p*-value < 0.05 in post hoc analysis (Bonferroni), between pre-test and follow-up. Abbreviations: M: mean; SD: standard deviation; MS: mean square; CG: Control Group; EG1: Experimental Group, 4 weeks; EG2: Experimental Group, 8 weeks; FFMQ: Five Facet Mindfulness Questionnaire; SCS: Self-Compassion Scale; JSPE: Scale of Physician Empathy.

**Table 4:** Comparison between groups in post-test scores, controlling pre-test scores, using ANCOVA.

Variable	Source	Type III Sum of Square	df	MS	F	*p*-Value	η^2^
**FFMQ**	FFMQ pre-test	10,240.20	1	10,240.21	46.396	<0.001	0.338
	CG/EG1/EG2	1443.94	2	721.97	3.271	0.042	0.067
	Error	20,084.98	91	220.71			
**SCS**	SCS pre-test	38.90	1	38.09	101.675	<0.001	0.528
	CG/EG1/EG2	4.53	2	2.26	6.046	0.003	0.117
	Error	34.09	91	0.37			
**JSPE**	JSPE pre-test	3400.18	1	3400.18	39.316	0.001	0.302
	CG/EG1/EG2	328.79	2	164.39	1.901	0.155	0.040
	Error	7870.04	91	86.48			

Abbreviations: df: degrees of freedom; M: mean; SD: standard deviation; MS: mean square; CG: Control Group; EG1: Experimental Group, 4 weeks; EG2: Experimental Group, 8 weeks; FFMQ: Five Facet Mindfulness Questionnaire; SCS: Self-Compassion Scale; JSPE: Scale of Physician Empathy.

**Table 5:** Comparison between groups in follow-up scores, controlling pre-test scores, using ANCOVA.

Variable	Source	Type III Sum of Square	df	MS	F	*p*-Value	η^2^
**FFMQ**	FFMQ pre-test	13,867.36	1	13,867.36	66.304	<0.001	0.456
	CG/EG1/EG2	687.25	2	343.63	1.643	0.200	0.040
	Error	16,522.68	79	209.15			
**SCS**	SCS pre-test	37.15	1	37.15	114.242	<0.001	0.591
	CG/EG1/EG2	2.52	2	1.26	3.880	0.025	0.089
	Error	25.69	79	0.32			
**JSPE**	JSPE pre-test	3259.28	1	3259.29	20.553	<0.001	0.206
	CG/EG1/EG2	732.76	2	366.38	2.310	0.106	0.055
	Error	12,527.60	79	158.58			

Abbreviations: df: degrees of freedom; M: mean; SD: standard deviation; MS: mean square; CG: Control Group; EG1: Experimental Group, 4 weeks; EG2: Experimental Group, 8 weeks; FFMQ: Five Facet Mindfulness Questionnaire; SCS: Self-Compassion Scale; JSPE: Scale of Physician Empathy.
